# Meeting of the Alumni Association of the Buffalo Medical College

**Published:** 1875-03

**Authors:** 


### Editorial.

### Meeting of the Alumni Association of the Buffalo Medical College.

The Association of the Alumni and officers of the Medical Department of the University of Buffalo, held its inaugural meeting at the Buffalo Medical College, Tuesday, February 23d. As most of our readers are aware an organization has been in existence ever since 1871, but it has not until the present time been deemed advisable to call a meeting of the Alumni. Within the past few months a committee having the matter in charge have procured articles of incorporation, and prepared a constitution for the adoption by the Association.

The meeting on the twenty-third was called to order by the Vice-President, under the old organization, Dr. W. W. Miner, who read the Certificate of Incorporation, which defines the object of the Association, etc., and names Drs. J. P. White, M. G. Potter, C. C. Wyckoff, D. E. Chace and Julius F. Miner, trustees for the first year.

This certificate was adopted as were also the constitution and by-laws which were read at the same time.

The following is a list of those who were present and became members of the organization:

W. King, J. E. King, 1847; C. C. Wyckoff, W. B. Gould, H. Hoyt, B. K. Stoddard, A. G. Ellinwood, 1848; F. F. Hoyer, 1849; W. W. Jones, L. G. Hall, John Root, 1850; J. C. Elliott, T. D. Strong, 1851; G. W. Whitney, 1852; C. L. Dayton, 1853; M. Baker, 1854; Geo. W. Barr, 1856; Henry Nichell, 1857; Thos Cushing, A. F. D. Jansen, 1858; W. H. Mason, W. W. Potter, 1859; W. Delos Hunt, C. H. Richmond, J. W. Robinson, 1860; T. M. Johnson, 1861; Edward Little, James S. Smith, 1862; S. W. Wetmore, 1863; J. M. Brown, P. A. Balcom, 1864; J. K. Griffin, Henry Lapp, John

H. Pickett, D. D. Loop, I. J. Whitney, A. H. Smith, 1865; M. B. Shaw, A. D. Ingham, W. C. Phelps, 1866; E. C. W. O'Brien, Wm. Martin, M. G. Potter, H. G. Hopkins, 1867; J. M. McWarph, H. B. Hall, D. E. Chace, F. E. Bliss, 1868; Geo. W. Pattisson, R. A. Patchin, E. G. Harding, F. A. Jones, M. B. Searls, 1869; H. B. Kibbler, R. Y. Charles, G. A. Wallace, W. O. Huggins, W. J. Cronyn, 1870; A. H. Briggs, W. W. Miner, 1871; F. E. L. Brecht, W. C. Raymond, 1872; D. C. Hunter, F. E. Dewey, D. H. Bailey, W. H. Slacer, A. T. Livingston, 1873; T. H. Boysen, J. A. Love, E. N. Brush, W. J. Howe, P. H. Quick, E. S. Weet, J. A. Pettitt, 1874.

After the adoption of the constitution a committee of three were appointed who named the following officers for the ensuing year, who were unanimously elected:

For President—W. W. Jones; 1st Vice President—A. G. Ellinwood; 2d Vice President—M. Baker; 3d Vice President—W. W. Miner; 4th Vice President—H. D. Vogsburg; 5th Vice President—J. B. Samo; Secretary—Wm. C. Phelps.

Executive Committee—The President, the Dean, ex-officio, E. N. Brush, A. H. Briggs, R. A. Patchin.

The retiring President, Dr. T. D. Strong, of Westfield, then delivered an address to the Association. He referred briefly to the organization of the Association, and congratulated the College that her sons had publicly recognized her claims, and had organized for the purpose of aiding her efforts to bestow good, sound professional education. He spoke of the stand which the College had taken toward the advancement of medical education, and said that she looked to the Alumni to support her in the effort. Referring to the efforts which had recently been made to insure the better qualification of students about entering the study of medicine; he said that the Colleges could not be expected to turn out graduates of a high order unless the material furnished was such as would admit it.

The address contained several other points of interest which we cannot at this time mention. At the conclusion of the President's address, the Association adjourned to assemble in the evening at St. James Hall to participate in the commencement exercises, and listen to the Alumni address by Dr. W. W. Potter, of the class of "59."

The inaugural meeting of the Association was very happily closed by a banquet at the Tifft House following the exercises at the Hall. The large dinning hall was filled by the officers, Alumni and invited guests of the College, and for a time medical topics were forgotten in the discussion of the contents of the tables. Upon the removal of the cloth, toasts were in order, and were severally announced by the President. With the exception of being somewhat lengthy, the responses were all appropriate, and frequently elicited marks of approval.

At a late hour the banquet closed and with it the first regular meeting of the Alumni. May many more as pleasant follow.

Lack of time and space prevents us from referring to several features of the organization which are worthy of attention, and to which a few remarks will be directed in a future number.

